# Editorial: Environmental and molecular control of bud dormancy and bud break in woody perennials: An integrative approach

**DOI:** 10.3389/fpls.2023.1104108

**Published:** 2023-02-22

**Authors:** Hisayo Yamane, Fernando Andrés, Songling Bai, Eike Luedeling, Etti Or

**Affiliations:** ^1^ Graduate School of Agriculture, Kyoto University, Kyoto, Japan; ^2^ UMR AGAP Institut, Univ Montpellier, CIRAD, INRAE, Institut Agro, Montpellier, France; ^3^ College of Agriculture and Biotechnology, Zhejiang University, Hanzhou, China; ^4^ Department of Horticultural Sciences, University of Bonn, Bonn, Germany; ^5^ Department of fruit tree sciences, Volcani Center, ARO, Rishon Lezion, Israel

**Keywords:** bud dormancy, bud break, climate change, tree phenology, modeling

Bud dormancy in woody perennials is essential for plant survival during winter (Cooke et al., 2012). It is also an important developmental stage that affects bud break, blooming, and new growth. Environmental cues (such as photoperiod, temperature and water status), genetic factors and horticultural practices (such as exogenous treatment of plant growth regulator), are all involved in the regulation of tree growth and the dormancy cycle. In recent decades, our understanding of bud dormancy and activity cycles in many plant species has increased substantially, while at the same time, diverse mechanisms behind bud dormancy and phenology-related processes (such as growth cessation and bud break) have become evident. In Rosaceae, for example, recent findings raised the possibility that multiple mechanisms underlie the behavior of vegetative buds and flower buds during the dormancy cycle (Yamane et al., 2021). In support, quantitative trait loci for vegetative and flower buds were differentially located in Japanese apricot (*Prunus mume*) (Kitamura et al., 2018). Moreover, temporal suspension of developmental arrest in sweet cherry (*P. avium*) flower buds was linked to the specific morphological stage regardless of the bud break capacity of dormant buds, which is generally linked to the vegetative bud dormancy depth (Fadón et al., 2018; Goeckeritz and Hollender, 2021; Yamane et al., 2021).

Among environmental cues, temperature signals have long been considered as the most important environmental factor controlling the progression of dormant buds towards bud break. Generally, Rosaceae fruit trees must receive sufficient exposure to cool conditions to fulfill their chilling requirements (CR) and then enough warmth to fulfill their heat requirements (HR) in order to show proper vegetative and flower bud break and regular bloom in the spring (Fadón et al., 2020). Hence, warming in fall, winter and spring due to climate change is expected to impact bud dormancy release, timing and intensity of growth resumption and bloom progression, all of which may threaten the stability of fruit and timber production and disrupt the sustainability of the ecosystem (Luedeling, 2012). While recent molecular studies have improved our understanding of the mechanisms that regulate and execute the bud dormancy cycle in woody perennials including fruit trees (Falavigna et al., 2019; Yang et al., 2021; Nilsson, 2022), a complete picture is still elusive. Thus, our ability to develop powerful and reliable means to control timing and intensity of dormancy, flower primordia development, bud break and bloom is still limited. Addressing this limitation requires an integrative approach that allows weaving together fragmented pieces of knowledge from various fields, such as genetics, physiology, breeding, meteorology, mathematical modelling and more. In accordance, the current Research Topic aims to (1) collect updated multifaceted knowledge regarding the dormancy cycle, growth resumption, and bloom progression, (2) allow future integration of such fragmented knowledge into a working model for reliable prediction of tree phenology responses to environmental changes during the tree dormancy period, including their implications for bloom timing and productivity. A reliable working model would be instrumental for optimizing breeding strategies and for developing horticultural manipulations aimed at adapting to higher winter temperatures, constituting a valuable contribution to the forestry and horticulture industries.

The Research Topic was launched in October 2020 and we have collected a 23 original research, method and review articles. We introduce some of these articles below.

## Proposed genes and pathways underlying bud dormancy and dormancy-related processes

Wu et al. reported that (1) abscisic acid (ABA) level decreased in *Magnolia wufengensis* buds during dormancy release (which was defined by a gradual increase in bud break rate using a bud break competency test under forcing conditions), (2) exogenous ABA (100 µM) application promoted dormancy maintenance (which was defined by bud break repression in the same test) and (3) RNA-seq data suggested that *MwCYP707A*, a key ABA catabolic gene, may be involved in dormancy release of *M. wufengensis.* Wang et al. reported on a comprehensive comparative analysis of seasonal changes in gene expressions and metabolites between high- and low-CR cultivars of herbaceous peony (*Paeonia lactiflora*). This analysis led the research group to propose an interesting model underlying the negative effects of warm winter climate on bud break. Noticeably, the authors speculated that accumulation of reactive oxygen species (ROS) affects ABA and bioactive gibberellin A3 (GA_3_). Lower GA levels in high-CR cultivars may result in blocking of the vascular tissue and abnormal bud development. These interesting hypotheses will require further studies to genetically test the biological relevance of the proposed model

Three research articles reported on the roles of poplar orthologs of Arabidopsis flowering regulators, *FD*, *FLOWERING LOCUS T* (*FT*) and *SHORT VEGETATIVE PHASE* (*SVP*), on dormancy-related vegetative growth traits, proposing that these genes control vegetative growth through the modulation of the GA metabolism. Sheng et al. generated *Populus FD-like3* (*FDL3*) overexpression lines, finding that FDL3 delayed short day-induced growth cessation. Gómez-Soto et al. generated *FT2* loss-of-function lines using CRISPR/Cas9, finding that *FT2* plays a dual role in poplar vegetative growth under long day conditions. FT2 promotes the GA 13-hydroxylation pathway and GA_1_ levels to sustain shoot apex development. However, in leaves, FT2 reduces the GA_1_ level by increasing *GA2 OXIDASE1* and decreasing *GA3 OXIDASE2* mRNA expression to restrict internode elongation. Earlier studies had proposed that *SVL*, an ortholog of *SVP* in poplar, controls temperature-mediated bud dormancy (Singh et al., 2018; Singh et al., 2019). Here André et al. reported that *SVL* also contributes to the regulation of short day-induced growth cessation and bud set through its expression in leaves. Moreover, *SVL* represses *FT2* transcription and decreases active GAs amounts by repressing the transcription of *GA20 OXIDASE*.

Another group of research articles highlighted genes that are highly expressed in winter dormant buds and proposed their relation to cold stress and blooming. Li et al. examined 33 AQUAPORIN (AQP) encoding genes in the apricot (*Prunus armeniaca*) genome. AQPs play a major role in plant water homeostasis and transportation of uncharged solutes across biological membranes. The research group found that *PLASMA MEMBRANE INTRINSIC PROTEIN1-3 (PaPIP1-3)*, *PaPIP2-1* and *TONOPLAST INTRINSIC PROTEIN1-1* (*PaTIP1-1*) were expressed in flower buds during winter, enhancing tolerance to cold stress when overexpressed in Arabidopsis. Lempe et al. (2022) analyzed expressions of 40 known candidate genes involved in regulation of apple (*Malus domestica*) dormancy using buds from trees grown under temperate climate conditions. Arabidopsis *PHYTOCHROME INTERACTING FACTOR4* (*PIF4*) regulates expression of *YUCCA8*, a key auxin biosynthesis gene (Sun et al., 2012) and acts as a crucial regulator of thermomorphogenesis (Franklin et al., 2011). Earlier research had reported that its apple orthologue *MdPIF4* presented changes in expression during dormancy release and is up-regulated in the winter (Takeuchi et al., 2018). Interestingly, (Lempe et al.) reported that the slope of the linear correlation of temperature with the expression of *MdPIF4*, analyzed from autumn to spring, differed between cultivars with contrasting blooming date, suggesting its potential role in regulation of cultivar-dependent inflorescence differentiation and blooming progression in apple.

## Candidate genetic components controlling dormancy-related traits: MADS-box transcription factors

Genetic resources with wide variation of dormancy-related traits such as early/late blooming date, high/low CR, deciduous/evergreen trait, can be instrumental not only as breeding materials but also for genetic analysis towards identification of important genetic factors that regulate these traits. The following research articles reported the results of genetic analysis on dormancy-related traits. Calle et al. used ‘Cristobalina’, an early blooming sweet cherry cultivar, to identify major QTLs controlling CR. In the peach *evergrowing* mutant that barely enters dormancy, four *DAM*s were deleted and the expressions of the remaining two *DAM*s were repressed (Bielenberg et al., 2008). Recent studies suggested that *DAM*s act as bud set promoters and bud break repressors, and a reduction of DAMs expression levels decreases CR in Rosaceae fruit crops (Yamane et al., 2019; Wu et al., 2021a). Calle et al. found that significant amino acid differences and structural mutations in *PavDAM*s in the major QTL were co-segregated with low-CR and early blooming in an F_2_ population. Interestingly, the mutations were conserved in a set of early blooming cultivars, suggesting that structural variation in these *DAM*s may be responsible for low-CR and early blooming. To identify genetic components controlling the evergreen/deciduous trait, Harel-Beja et al. used several evergreen pomegranate (*Punica granatum*) accessions from different genetic resources in the Israeli pomegranate collection. Fine mapping with F_3_ and F_4_ populations from several segregating populations revealed that mutation in a gene encoding a unique MADS transcription factor (mostly similar to Arabidopsis *AGL27*), called *PgPolyQ-MADS*, is responsible for the evergreen trait. Interestingly, the polyQ domain (containing a repeat of eight glutamines) of PgPolyQ-MADS resembles that of the ELF3 prion-like domain, which was recently reported to act as a thermosensor in Arabidopsis (Jung et al., 2020), suggesting that *PgPolyQ-MADS* may mediate the response to temperature changes through DNA binding and control leaf shedding. Collectively, these reports further support the involvement of genes encoding MADS-box transcription factors as putative genetic factors underlying dormancy-related traits. Moreover, the identified genes and genomic regions can be used as molecular markers for efficient breeding of phenology-related traits (such as low-CR) towards the development of new cultivars that are adapted to climate change.

## Modeling temperature effects on dormancy and bud break: Key challenge in predicting impacts of global climate change on tree phenology

Since bud break and bloom timing can theoretically be determined by the (largely) sequential fulfilment of CR and HR, construction of models predicting genotype-specific CR and HR is an important task. Precise models can provide the grower with valuable information that will improve decision-making regarding cultivar selection and cultural practices. They can also help predict the suitability of a given location under predicted future climate condition and thus help mitigate the risk of future negative impacts of climate change on tree growth and the dormancy cycle. The following research articles addressed this important issue. An interesting study by Bielenberg and Gasic revealed the genetic variation of HR, independently of CR. In peach, selection of phenotypes with improved spring frost avoidance can be achieved through breeding with delayed-bloom genotypes. The authors proposed that selecting cultivars that require more growing degree hours (GDH) would be a safer, more reliable strategy for delaying bloom than choosing cultivars with increased base temperature for GDH accumulation. In addition to meteorological data, reliable associated physiological and morphological biomarkers are essential to allow precise determination of CR and HR. del Barrio et al. proposed that decrease in sucrose content in dormant buds could serve as a reliable biological marker for the completion of CR fulfillment in Persian walnut (*Juglans regia*). Herrera et al. concluded that male meiosis could be a biomarker to determine CR fulfillment timing in apricot based on their long-term measurements.

We also collected 2 review articles, both of which focus on the adverse effects of warming on bud dormancy progression and bud break. Tominaga et al. summarized studies on flowering (dormancy) disorders in Japanese pear (*Pyrus pyrifolia*), concluding that warm temperatures during autumn and winter result in flowering disorders through their interfering effects on cold acclimation, dormancy progression and floral bud maturation. They raised the hypothesis that the amounts of chilling exposure required for dormancy completion (floral bud maturation) and dormancy release (release from the repression of bud break) could be different and independently regulated, at least partially. Guillamón et al. provided a summary of the currently available agrochemicals that can compensate the incomplete fulfillment of CR. They described the current knowledge available for the well-established and most efficient agrochemical, hydrogen cyanamide, and discussed the need for caution in light of potential phytotoxic effects recorded in various studies, where dormancy status, environmental conditions and species/cultivar sensitivity are key parameters in assessing such toxicity. While the authors introduced several available alternative agrochemicals, including nitrogen-based compounds, they stressed that new environmentally friendly agrochemicals should be developed through deeper foundational and applied research activities.

## Future prospects

Increased activity in recent years in the fields of cellular and molecular aspects of bud dormancy, which reflect an increase in the number of research groups and plant species, was accompanied by realizations of the community regarding the importance of reexamining the definition and terminology of “dormancy” (Considine and Considine, 2016; Yamane et al., 2021). For example, recent analyses of events during the dormancy cycle in grapevine buds led Shi et al. (2020) to propose a working hypothesis that addresses the potential “Core of dormancy”. According to that hypothesis, auxin, cytokinins, *FT* and GA may participate in meristem reactivation rather than in dormancy release. The working model also proposed (1) that an “energy crisis, starvation, and starvation-triggered catabolism cascade” may act at the heart of the dormancy phase establishment and release, and (2) that interplay around the Snrk : TOR (Sucrose non Fermenting related kinase: target of rapamycin kinase) basic regulon may serve as a critical component in regulation of transition into and out of the dormancy phase, *via* regulation of both macromolecule catabolism and cell cycle. If cell division appears to be a reliable index of dormancy, a methods paper in this Research Topic may become helpful. Hermawaty et al. proposed a protocol that will allow to estimate the cell cycle status of dormant grapevine (*Vitis vinifera*) buds through optimization and validation of flow cytometry data analysis. This protocol can be applied to find out if division of cells of a dormant meristem is arrested in the G1 phase – an open question that has been extremely difficult to address so far.

It is our understanding that a collaborative effort of the community to revisit and refine the definitions that are related to the process of bud dormancy is of great value, due to its potential to better focus and better integrate research targets and findings towards understanding “what is the core of bud dormancy”. Another critical future focus is dictated by bud dormancy being an important agronomic trait, for which regulation by artificial agents is crucial in certain growing regions and may become relevant for other regions in the future. In this context, understanding the effects of chill accumulation on flower primordia development and seasonal growth regulation (which we may describe as “periphery of dormancy”) is a research target of equal importance to that of understanding the nature of the “core of dormancy”. In summary, these are both important and attractive issues in horticulture with a complementary nature, since both bud break/blooming following bud dormancy and bud dormancy itself can be a target for manipulation (Goeckeritz and Hollender, 2021).

With this in mind, the 7^th^ Plant Dormancy Symposium, which will be held in Perth, Australia in September 2023, will provide an excellent opportunity for the dormancy community to discuss the terminology, share research findings and exchange ideas regarding the “core of dormancy”, and hopefully to establish new scientific collaborations.

Finally, as the editors of this Research Topic, we wish to thank all the authors who have contributed articles to this collection. We also wish to express our appreciation and thanks to all the reviewers for their generous support and valuable comments that helped to improve the quality of the manuscripts. We sincerely hope that this Research Topic will be useful for the scientific and industrial communities and will supply researchers in the bud dormancy research community with tools and information that may help to design their future research.

## Author contributions

All authors listed above made a substantial contribution to the work and approved it for publication.
